# Wild food plants traditionally consumed in the area of Bologna (Emilia Romagna region, Italy)

**DOI:** 10.1186/1746-4269-10-69

**Published:** 2014-09-25

**Authors:** Sabrina Sansanelli, Annalisa Tassoni

**Affiliations:** Department of Biological, Geological and Environmental Sciences, University of Bologna, Via Irnerio 42, 40126 Bologna, Italy

**Keywords:** Ethnobotany, Traditional local knowledge, Wild food plants, Bologna, Emilia-Romagna region, *Crepis vesicaria* subsp. *taraxacifolia*, *Urtica* spp

## Abstract

**Background:**

This research was performed in an area belonging to the province of the city of Bologna (Emilia-Romagna region, Northern Italy). The purpose of the present survey was to record the local knowledge concerning traditional uses of wild food plants and related practices, such as gathering, processing, cooking, therapeutic uses, with the aim of preserving an important part of the local cultural heritage.

**Methods:**

Thirty-nine people still retaining Traditional Local Knowledge (TLK) were interviewed between March-April 2012 and September - October 2013 by means of open and semi-structured ethnobotanical interviews. For each plant species mentioned, we recorded the botanical family, the English common name, the Italian common and/or folk names, the parts of the plant used, the culinary preparation, and the medicinal usage. The relative frequency of citation index (RFC), a tool that measures the local cultural importance of a plant species, was also included.

**Results:**

The folk plants mentioned by the respondents belonged to 33 botanical families, of which the Rosaceae (14 plants) and the Asteraceae (9 plants) were the most representative. The species with the highest RFC index (0.77) were *Crepis vesicaria* subsp. *taraxacifolia* (Thuill) Thell and *Taraxacum officinale* Weber. Eleven folk plants were indicated as having therapeutic effects. *T. officinale* Weber, *C. vesicaria* subsp. *taraxacifolia* (Thuill) Thell and *Sonchus* spp., which are used as food, were reported to be depurative, blood cleaning, refreshing, diuretic and laxative. The most commonly used species was *Urtica* spp, which was also the most frequently cited for medicinal uses.

**Conclusions:**

The present survey documented the wild food plant traditional knowledge of an area belonging to the province of the city of Bologna (Emilia-Romagna region, Northern Italy). The general perception obtained is that on one side the TLK related to wild food plants has strongly been eroded, mainly due to immigration and urbanization phenomena, whereas on the other side these plants are revaluated today because they are perceived as healthy and also because they represent the preservation of biodiversity and a way of getting back to nature.

**Electronic supplementary material:**

The online version of this article (doi:10.1186/1746-4269-10-69) contains supplementary material, which is available to authorized users.

## Background

Before the so-called *economic boom* (1950–1970), Italy was mainly an agriculture-based economy and society. Poverty, dryness and wars made it difficult to meet subsistence needs [1] and, therefore, edible wild plants represented an alternative food source or sometimes the only one [2]. Wild food plant gathering practices and their way of consumption were slowly integrated into the customs of a territory, becoming part of the Traditional Local Knowledge (TLK). The process of industrialization and urbanization changed the way of living and society, which became less and less rural. The use of mechanized agriculture and the development of transport improved the availability of vegetables and, consequently, wild food plant practices and the related local knowledge, strongly connected with rural societies, almost totally disappeared. Furthermore, intensive agriculture, which generally involved extensive use of pesticides, and pollution largely impaired wild flora biodiversity, reducing the availability of some wild plants used as food in the past.

The majority of ethnobotanical research has been preferentially focused on traditional medicinal plants [3, 4], giving less attention to wild food plants, however, over the last two decades, an increasing interest in wild food plants, even in modern societies, has led to many local ethnobotanical studies [5–7]. The international political attention towards biodiversity topics and its links to nutrition and health (Convention on Biological Diversity in 1992, Year of Biodiversity in 2010) has surely contributed in driving forward wild food plants research. Several researches demonstrated that many edible wild plants have nutritional or therapeutic value due to the presence of biologically active compounds and, therefore, they can be considered as food-medicine [8–10]. For example *Tamus communis* and *Humulus lupulus* contain a high amount of, respectively, citric and malic acids, antioxidants which are beneficial to health due to their ability to chelate metals [8]; *Borago officinalis* resulted to be a source of γ-linoleic acid and other fatty acids that are precursors to mediators of the inflammatory response [9]; *Raphanus raphanistrum* showed anti-diabetic and anti-proliferation activities while *Cynara cardunculus* demonstrated a high mood-disorder regulating activity [10].

Wild food plants are generally characterized by high nutritional and low energy values [11]. In comparison to the corresponding cultivated species, wild food plants have a higher fibre content [12], are rich in antioxidants and flavonoids [13] and contain very low amounts of lipids [11]. Many were proven to have important beneficial effects in preventing several chronic diseases of modern society, such as age-related and heart pathologies, diabetes and some types of cancer [10, 11, 14, 15]. In the Mediterranean area, the use of wild food plants was thoroughly investigated during the years 2003–2005 by the European Union-funded RUBIA Project [16]. The selected study sites were Albania, Cyprus, Egypt, Greece, Italy, Morocco and Spain, countries in which the way of using wild plants, closely related to traditions, environment and cultural heritage, varied greatly. Although the most reported species were sometimes the same (e.g. belonging to Asteraceae and Lamiaceae families), their cultural importance varied among the different areas. The habit of using wild food plants played an important role in the life of Mediterranean rural people, however, the spread of plant folk uses has been progressively decreasing over the last generations, and is particularly evident in urban areas [16, 17]. In Italy, a comparative ethnobotanical study on wild food plants analysing twenty-one communities located throughout the Italian peninsula, including the islands of Sardinia and Sicily, produced a comprehensive picture of the country [18]. This survey showed several differences in the use of wild plants, with few botanical species mentioned in more than one area and in particular *Borago officinalis* present in both Southern and Northern Italian sites. The most important difference was the prevalence of the Rosaceae family in the North, while species belonging to Asteraceae, Brassicaceae and Liliaceae were most frequently cited in the South of Italy. In general, the results showed that in Southern Italy the erosion of wild TLK plants was happening at a slower rate than in Northern Italy [18].

Changes in the contemporary use of wild food plants in Italy and other European countries have also been recently studied [19]. The results confirmed that the traditional use of wild edibles has been steadily decreasing in association with new phenomena appearing in modern societies, such as the presence of new ethnic minorities that maintain their own traditions and food habits [19].

The study area of the present survey comprised part of the territory of the province of Bologna located in the Emilia-Romagna region (Northern Italy), one of the more economically developed regions of Italy. In this area, after the end of Second World War, many socio-economic changes occurred bringing economic well-being, industrial activities and the development of new transport infrastructures that well connected people, houses, services and workplaces. These changes inevitably mutated the lifestyle, the family system and the nature of communities. The local knowledge, shared among family and community members, was thus less and less passed down to the following generations. The aim of the present paper is to record local knowledge concerning the traditional uses of wild food plants as well as related practices such as gathering, processing, cooking and therapeutic uses. Up till now no research has been carried out on the use of wild food plants in this territory and, therefore, this study represents the first attempt to collect and save from oblivion an important part of the cultural heritage preserved by this population.

## Methods

**Figure 1:**
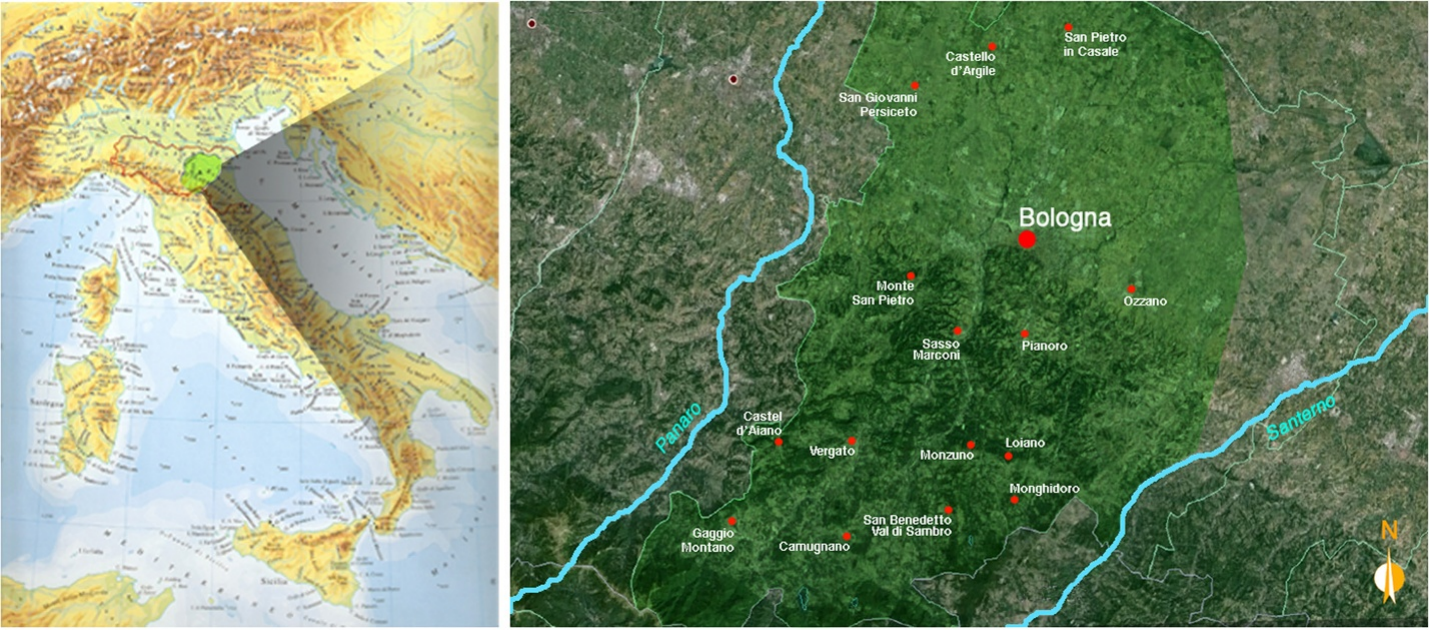
**Location of the study area.**

Fieldwork was conducted in a study area belonging to the Bologna province (Emilia-Romagna region, Northern Italy) comprised between the Panaro river (to the north-west), the Santerno river (to the south-east), the Ferrara province (to the north*-*east), the Apennine mountains (to the south*-*west) (Figure 1). The survey was performed during the following periods: March-April 2012 and September - October 2013. Ethnobotanical information was collected by standard ethnobotanical tools [20], such as participant observation, as well as open and semi-structured interviews. A questionnaire form, used as a guideline for the ethnobotanical interviews, is reported in the Additional file 1. Thirty-nine people still retaining Traditional Local Knowledge (TLK) were interviewed. The respondents were identified after having contacted several local associations for elderly people. All participants as well as their parents were born and had always lived in the study area (Figure 1). The origin of the family home is of extreme importance as TLK is formed and handed down mainly within the family. However, it was not easy to find people who satisfied these selection criteria, as the province of Bologna is an area with a high rate of immigration and a high level of urbanization that presumably greatly contributed to the loss of TLK. Conversations and discussions were also made with people working in vegetarian restaurants, organic farms and botanical gardens to obtain information on the actual use and knowledge of wild food plants. The purpose, method and nature of the research were previously explained and informed consent was obtained from all informants. Interviews were carried out both individually and in groups. When conducted in groups, the respondents were stimulated to express their personal experience. During the first phase of the interview, the informants were asked to freely recall all the wild plant species that they had used in the past and/or were presently using for food purposes. For each plant species mentioned, the informants were asked to state the folk name, the parts of plant used, the period of harvesting, culinary and other possible uses, the frequency of use and whether they had used the plant in the past or were still using it. Processing and cooking activities were also precisely described. The respondents were able to speak freely but sometimes it was necessary to encourage them providing some suggestions (e.g. *“have you ever seen/used this plant?”*) just to help them to recall the memories of decades ago. Particular attention was paid to the therapeutic effects that may have been perceived after ingestion of some particular wild food plant. Moreover, the medicinal use of plants, with specific modes of preparation and application, were always addressed. The perception of wild species in relation to their cultivated analogues and the possible impacts, benefits or risks on human nutrition and health, were also investigated. The taste and level of appreciation of the consumed plant species were described.

For each mentioned plant species a relative frequency of citation index (RFC) was calculated. The RFC index expresses the number of informants who cited a specific wild food plant divided by the total number of informants. It was used to assess the local importance of each species and may vary from 0, when nobody refers to the plant as useful, to 1, when all informants mention the use of a species [21]. Three informants who were particularly knowledgeable on wild food plants and still using them, were chosen as *key informants* to become involved in participant observations and their interviews were implemented to better understand the way of plant collection, food preparation, gender relation and mode of passing down local knowledge. The *key informants* were also helpful and active in gathering the mentioned wild food plant species, which they called by the relative folk or Italian common names. The collected wild plants specimens were successively identified by expert botanists (Dr. Mossetti Umberto and Dr. Managlia Annalisa of the University of Bologna), and renamed following standard botanical nomenclature [22]. To find the correspondence between folk and scientific names, a booklet regarding the names of plants used in the popular tradition of Bologna [23] resulted to be very useful to address and speed up botanical identification. Voucher specimens of the wild food plant species were collected and deposited in the Herbarium of the University of Bologna.

## Results and discussion

### Informants

Thirty-nine people, 25 women (64%) and 14 men (36%), were interviewed. The age of the informants ranged between 48 and 92, with a mean of 71 and a median of 75. Nine informants were younger than 60, 10 were aged between 60 and 75, and 20 were older than 75. Finding persons who still retained TLK about wild food plants uses was particularly difficult. This shows that much of the local knowledge has already been lost and that it is necessary and urgent to carry out this kind of research in this territory. The results showed that TLK was almost equally shared between the two genders (the average number of species quoted per gender was: 10.2 for women and 9.1 for men), however, the women gave much more details and information on the traditional wild food plants used. Women had a better preserved memory of it probably because the processing and cooking of wild plants were almost exclusively done by them while gathering activities were carried out by both genders. These data are partially in agreement with several studies performed in the Mediterranean area [24–26] that showed that women are the major depositaries of wild plant local knowledge.

Many conversations with people working in vegetarian restaurants, organic farms and botanical gardens led to understand that the traditions about wild food plants were very little known and shared among local population. However there is a part of society that is very careful about healthy and genuine diet and so very interested toward wild food plants (independently from their popular traditions) because considered rich of healthy components and, as study area is a highly anthropomorphized environment, a way to get closer to nature.

### Wild food plant data

The informants mentioned a total of 66 wild food plants (Table 1), including greens (leafy plants eaten as vegetables), fruits and semi-wild plants. The mean number of species quoted per informant was 9.8. Wild plants used for making liqueurs (in particular digestive liqueurs) were also taken into consideration, because these are traditionally drunk at the end of a meal. The wild edible plants mentioned are reported in Table 1 which lists the botanical species and family name, English and Italian common names, Italian folk names (when available), the parts of the plant used, the culinary and medicinal usage and the RFC. Most of the recorded species are commonly used in the Mediterranean area, such as *Cichorium intybus* L., *Sonchus asper* L., *Borago officinalis* Weber, *Papaver rhoeas* L. [12] (Table 1), whereas others are mainly eaten in Northern and Central Italy, such as *Bellis perennis* L., and *Capsella bursa-pastoris* (L.) Med. [18]. The RFC ethnobotanical index indicates, for a given folk species and analysed area, the degree of knowledge shared among the informants. The RFC may vary from 0 to 1, consequently, a RFC value close to 1 indicates that a species is very important from a cultural and traditional point of view. The highest RFC index (0.77) was found for *Crepis vesicaria* subsp. *taraxacifolia* (Thuill) Thell and *Taraxacum officinale* Weber. *C. vesicaria* subsp. *taraxacifolia* (Thuill) Thell is known by the folk name *strecapugno*, while *T. officinale* Weber is known as *piscialét. C. vesicaria* subsp. *taraxacifolia* (Thuill) Thell is more appreciated than *T. officinale* Weber because of its bitter, slightly crisp flavour, the memory of which is well preserved, even in people who do not consume it anymore. Moreover, *T. officinal*e Weber is a species widely known in Italy and abroad, not only as an edible plant, but also for its therapeutic properties used for depurative and digestive purposes and for mitigating hepatic diseases. Its high availability and characteristic ripe fruits make the plant easy to find and to collect. On the contrary, *C, vesicaria* subsp. *taraxacifolia* (Thuill) Thell, as revealed by the people interviewed, is not easy to recognise and quite difficult to find. The subspecies *taraxacifolia* of *C. vesicaria* is also consumed in other areas throughout Central Italy, often as a substitute for *C. vesicaria* L. subspecies *vesicaria* and *Crepis biennis* L. [27]. *C. vesicaria* L. subspecies *vesicaria* is also present in the study area but people identify, collect and consume only the subspecies *taraxacifolia*. No information is available on knowledge and collection of *C. biennis* L.*.*

**Table 1:** **List of the wild food plants used in the study area**

Botanical name	RFC	Botanical family	English common name	Italian common and/or folk names	Parts of the plant used	Culinary use	Medicinal use (preparation and administration)
Achillea ptarmica L.*	0.03	Asteraceae	sneezewort	achillea	flowers	salads	
Allium schoenoprasum L.*	0.03	Liliaceae	chives	erba cipollina	leaves	flavouring	
Allium ursinum L.*	0.03	Liliaceae	wild garlic	aglio selvatico	bulbs	flavouring	
Asparagus acutifolius L.	0.15	Asparagaceae	wild asparagus	asparago selvatico/*asparagina*	shoots	salads, pan-fried	
Bellis perennis L.*	0.03	Asteraceae	common daisy	margheritina	young leaves	salads	
Borago officinalis L.	0.08	Boraginaceae	starflower	borragine	leaves	salads, pancakes, pies	
Calamintha nepeta L.*	0.03	Lamiaceae	lesser calamint	nepetella	leaves	salads	
Calendula officinalis L.*	0.03	Asteraceae	common marigold	calendula	flowers	salads	
Capsella bursa pastoris L.*	0.03	Brassicaceae	shepherd’s-purse	borsa del pastore	young leaves	salads	
Castanea sativa Mill.	0.10	Fagaceae	chestnut	castagno	fruits	fresh fruits	
Cichorium intybus L.	0.38	Asteraceae	wild chicory	cicoria selvatica/ *radećć, radećć cavdagn*	young leaves	salads, pan-fried	
Clematis vitalba L.	0.51	Ranuncolaceae	traveller’s joy	vitalba/*vizeibra*	shoots	salads, pan-fried, omelettes, mixed vegetables	
Cornus mas L.	0.05	Cornaceae	cornelian cherry	corniolo	fruits	rural snack, liqueurs	
Corylus avellana L.	0.05	Corylaceae	common hazel	nocciolo	fruits	fresh fruits	
Crataegus azarolus L.*	0.03	Rosaceae	azarole	azzeruolo/*lazaren*	fruits	rural snack	
Crataegus monogyna Jacq.	0.15	Rosaceae	common hawthorn	biancospino/*spen bianc*	fruits, shoots, leaves	rural snack	Relaxing, insomnia and heart problems (flowers infusion)
Crepis sancta (L.) Babc.	0.15	Asteraceae	hawk’s-beard	radicchiella/*ciocapiat*	young leaves	salads, pan-fried	Diuretic and laxative (food)
Crepis vesicaria subsp. taraxacifolia (Thuill.) Thell.	0.77	Asteraceae	beaked hawk’s beard	radicchiella/*strecapugno*	young leaves	salads, pan-fried, omelettes, pasta dough	Depurative, refreshing, blood cleaning, diuretic, laxative (cooking water, food)
Diplotaxis tenuifolia L. (DC)	0.38	Brassicaceae	wall rocket	rucola selvatica	leaves	salads	
Fagus sylvatica L.*	0.03	Fagaceae	common beech	*al fasoli (fruits)*	fruits	rural snack	
Ficus carica L.	0.05	Moraceae	common fig	fico	fruits	fresh fruits	
Foeniculum vulgare L.	0.13	Apiaceae	wild fennel	finocchio selvatico	stems (I), leaves (II), seeds (III)	liqueurs (I), flavouring (II, III), mixed vegetables (I)	
Gentiana lutea L.*	0.03	Gentianaceae	great yellow gentian	genziana	roots	liqueurs	
Humulus lupulus L.*	0.03	Cannaboideae	common hop	luppolo	shoots	pasta sauce	
Juglans regia L.	0.10	Juglandaceae	walnut	noce	fruits	liqueurs, fresh fruits	
Juniperus communis L.	0.15	Juniperoideae	common juniper	ginepro	fruits	flavouring, liqueurs	
Laurus nobilis L.	0.10	Lauraceae	bay laurel	alloro	leaves	flavouring, liqueurs	
Lippia citriodora Kuntze	0.08	Verbenaceae	lemon verbena	erba luigia	leaves	liqueurs	Swelling trauma (decoction)
Lonicera caprifolium L.	0.08	Caprifoliaceae	sweet honeysuckle	caprifoglio/*ligabôsc*	shoots	salads	
Malus sylvestris (L.) Mill*	0.03	Rosaceae	European crab apple	melo selvatico	fruits	fresh fruits	
Medicago sativa L.*	0.03	Fabaceae	alfalfa	erba medica/*spagna*	leaves	salads, mixed vegetables	
Melissa officinalis L.*	0.03	Lamiaceae	lemon balm	melissa, erba limone	leaves	flavouring	
Mentha spp.	0.13	Lamiaceae	mint	menta	leaves	flavouring, liqueurs	Digestive (decoction)
Mespilus germanica L.	0.05	Rosaceae	medlar	nespolo	fruits	fresh fruits	
Morus spp.	0.05	Moraceae	mulberry	mora	fruits	fresh fruits	
Papaver rhoeas L.	0.05	Papaveraceae	field poppy	papavero/*rosetta*	young leaves	salads, pan-fried, mixed vegetables	
Portulaca oleracea L.	0.05	Portulacaceae	purslane	portulaca	leaves	salads, liqueurs	
Primula spp.	0.10	Primulaceae	primrose	primula	flowers (I), leaves (II)	salads (I), rural snacks (I), pasta stuffing (II)	
Prunus avium L.	0.05	Rosaceae	wild cherry	ciliegio selvatico	fruits	fresh fruits	
Prunus cerasifera Ehrh.	0.10	Rosaceae	myrobalan plum	mirabolano/*rustican*	fruits	rural snacks	
Prunus cerasus L.	0.05	Rosaceae	sour cherry	amareno/*visciole*	fruits (I), leaves (II)	fresh fruits (I), liqueurs (II)	
Prunus laurocerasus L.	0.05	Rosaceae	lauroceraso	lauroceraso/*lauro*	fruits	liqueurs	
Prunus spinosa L.	0.28	Rosaceae	sloe	prugnolo selvatico/*prugnól, spini, strozchi*	fruits	rural snacks, liqueurs	
Punica granatum L.*	0.03	Punicaceae	pomegranate	melograno	fruits	fresh fruits	
Pyrus pyraster Burgsd	0.05	Rosaceae	wild pear	pero selvatico	fruits	fresh fruits	
Robinia pseudoacacia L.	0.21	Fabaceae	black locust	acacia/*acâg*	flowers	pancakes, rural snacks	
Rosa spp.	0.28	Rosaceae	dog rose	rosa selvatica*/pizzincul (fruits)*	shoots (I), fruits (II), flowers (III)	rural snack (I, II), jams (II, III)	
Rosmarinus officinalis L.	0.13	Lamiaceae	rosemary	rosmarino	leaves	flavouring, liqueurs	Digestive (decoction); decongestant (fumigations)
Rubus spp.	0.31	Rosaceae	wild blackberry	rovo/*râza*	fruits (I), shoots (II)	fresh fruits (I), liqueurs (I), jams (I), rural snacks (II)	
Rumex acetosa L.	0.15	Polygonaceae	sorrel	acetosa/*êrba brossca*	leaves	rural snacks	
Ruscus aculeatus L.*	0.03	Ruscaceae	butcher’s broom	pungitopo	shoots	pan-fried	
Salvia pratensis L.	0.10	Lamiaceae	meadow clary	salvia selvatica	leaves	salads, flavouring, omelettes, liqueurs	Digestive (decoction); female genital problems (infusion); toothpaste (fresh leaves)
Sambucus nigra L.	0.21	Adoxaceae	elderberry	sambuco	fruits (I), flowers (II)	jams (I, II), pancakes (II), liqueurs (II)	Antirheumatic (food: jam)
Sanguisorba minor Scop.	0.05	Rosaceae	salad burnet	pimpinella/*pampinela*	leaves	salads	
Satureja hortensis L.*	0.03	Lamiaceae	summer savory	santoreggia	leaves	flavouring	
Silene vulgaris (Moench) Garcke	0.26	Caryophyllaceae	bladder campion	silene rigonfia/*strìdel, ciucchett*	young leaves	salads, pan-fried, pasta sauce, pasta dough, omelettes	
Sonchus asper L. (Hill)/Sonchus arvensis L.	0.33	Asteraceae	sow thistles	grespino/*frabbs*	young leaves	pan-fried, salads	Depurative, diuretic, laxative (food)
Sorbus domestica L.	0.13	Rosaceae	seviceberry	sorbo	fruits	fresh fruits	
Tanacetum balsamita L.*	0.03	Asteraceae	costmary	erba di santa Maria	leaves	liqueurs	
Taraxacum officinale Weber	0.77	Asteraceae	swines snout	tarassaco/*piscialet*	young leaves	salads, pan-fried, omelettes, mixed vegetables	Depurative, refreshing, draining, diuretic (food, cooking water)
Thymus spp.	0.08	Lamiaceae	thyme	timo	leaves	flavouring	
Trifolium pratense L.*	0.03	Fabaceae	red clover	trifoglio dei prati	flowers	rural snacks	
Urtica spp. (dioica, urens)	0.74	Urticaceae	nettle	ortica	leaves	pasta stuffing and dough, salads, omelettes, mixed vegetables	Refreshing, kidney problems, mineralizing (food, cooking water); hair strength, shine, dandruff (cooking water); anti- arthritic (fresh leaves rubbed on the body); insecticide (leaves macerated in water)
Valerianella locusta L. (Laterrade)	0.41	Valerianaceae	lamb’s lettuce	valerianella/*grassagallina*	leaves	salads	
Viola spp.	0.08	Violaceae	violet	viola	flowers	salads	
Vitis vinifera L. subsp. Sylvestris (Gmelin) Hegi	0.13	Vitaceae	wild grape	vite selvatica	fruits (I), shoots (II), leaves (III)	fresh fruits (I), rural snacks (II), mixed vegetables (III)	

Folk Italian names are written in italics.

Roman numbers indicate the correlation between the traditional culinary use and a specific part of the plant.

*indicates plant species mentioned by a single informant.

RFC: Relative Frequency of Citation Index.

Medicinal use: in brackets the way plants or parts of it are prepared and administered to give the mentioned therapeutic effect.

Other wild plants which scored a high RFC index value were: *Urtica* spp. (0.74), *Clematis vitalba* L. (0.51), *Valerianella locusta* L. (Laterrade) (0.41), *C. intybus* L. and *Diplotaxis tenuifolia* L. (DC) (0.38) and *Sonchus* spp. (0.33) (Table 1).

In particular, *Urtica* spp. resulted to be the most consumed species and is much more valued today than it was in the past. This plant is in fact well integrated in homemade local cooking (e.g. to make green pasta, or to fill and season hand-made pasta) and dishes containing *Urtica* are proposed by several local restaurants. The wide use of *Urtica* may also depend on the fact that is a ruderal plant, characterized by a rapid growth close to people’s residences, thus being readily available for collection and consumption.

In the present survey, herbs used to make hot beverages (decoctions or infusions) such as *Malva sylvestris* L. and *Matricaria chamomilla* L. were not considered as foods (not included in Table 1), but classified as medicinal plants having therapeutic effects.

A large group of plant species listed (19), were mentioned by a single informant for this reason they may be considered as uncertain data (Table 1). These results point out how strongly eroded is the knowledge about wild food plants in the study area. The data obtained in the present study were compared with an ethnobotanical survey conducted around Lake Vrana (northern Dalmatia, Croatia) [28] based on a similar number of informants (43) and mentioned wild food plants (57). Wild vegetables are still widely used in the Lake Vrana area. In fact, although less popular among young people, old and middle aged people still retain wide knowledge and collect them. The average number of species quoted is higher in Lake Vrana area (12.4) than in Bologna’s territory (9.8) on the contrary to the percentage of plant species mentioned by a single informant (14% for Lake Vrana study and 29% in the present study). This comparison confirms the highly eroded nature of wild food plants knowledge in the area of Bologna.

**Figure 2:**
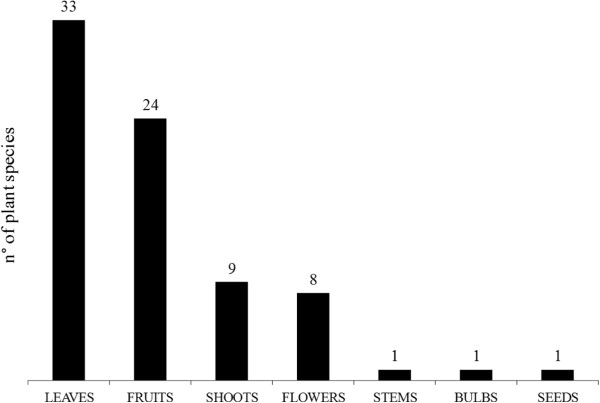
**Parts of the wild food plants traditionally consumed in the study area.** The number above each bar indicates the total number of species used in each category.

**Figure 3:**
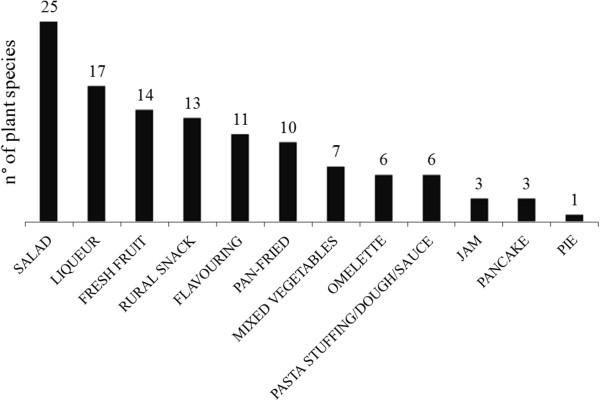
**Culinary uses of the wild food plants traditionally consumed in the study area.** The number above each bar indicates the total number of species used in each category.

The folk plant species mentioned by the people interviewed in the present study belonged to 33 different botanical families (Table 2). The most representative families were the Rosaceae (14 plants) and the Asteraceae (9 plants). The parts of the plants used and recorded for each mentioned species are represented in Figure 2. In general, leaves were most frequently used (33), followed by fruits (24) and shoots (9). The ways of consumption of wild food plants and the number of species in each category are shown in Figure 3. Plants were most often consumed raw, in salads prepared with the tender young leaves (25) collected in the early vegetative *rosetta* stage when they have a less bitter taste, or boiled. They are also frequently used as liqueur ingredients (17), a habit still in use, or eaten as fresh fruits (14). In the past, some wild plant parts were extemporaneously eaten raw as a rural snack (13, Figure 3) by kids and collecting them was often experienced as a competing game. Rural snacks consisted mainly of berries, but also of young shoots, leaves and flowers, such as those of *Primula* spp. L., *Trifolium pratense* L and *Robinia pseudoacacia* L., from which, in particular in case of the latter, the sweet nectar was sucked. In addition to flowers and ripe fruits, kids were often attracted by the sour taste of unripe wild fruits and young shoots. The only rural snack consumed as leaves was *Rumex acetosa* L. popularly known as “*erba brossca”* which means in fact sour grass.

**Table 2:** **Botanical families of wild food plants traditionally consumed in the study area**

Botanical family	N° of wild food plant species
Rosaceae	14
Asteraceae	9
Lamiaceae	7
Fabaceae	3
Brassicaceae	2
Fagaceae	2
Liliaceae	2
Moraceae	2
Adoxaceae	1
Apiaceae	1
Asparagaceae	1
Boraginaceae	1
Cannaboideae	1
Caprifoliaceae	1
Caryophyllaceae	1
Cornaceae	1
Corylaceae	1
Juniperoideae	1
Gentianaceae	1
Juglandaceae	1
Lauraceae	1
Papaveraceae	1
Polygonaceae	1
Portulacaceae	1
Primulaceae	1
Punicaceae	1
Ranuncolaceae	1
Ruscaceae	1
Urticaceae	1
Valerianaceae	1
Verbenaceae	1
Violaceae	1
Vitaceae	1

In general, the Asteraceae wild greens were cooked by pan-frying or consumed together with other wild plants as mixed vegetables (Figure 3 and Table 1).

The plant species of the present study (area of Bologna, northern Italy) were compared with those listed by two Italian ethnobotanical surveys focused in wild food plants traditions and carried out in Castelmezzano village and in the Graecanic area (Lucania and Calabria region, southern Italy) [25, 26]. From a general point of view, among the three study areas most of the recorded plant species are common, like most of the wild fruits and some Asteraceae plants. On the other hand, some specific differences could be pointed out such as for thistles (*Carlina acaulis* L., *Cynara cardunculus* L. ssp. *cardunculus*, *Silybum marianum* L., *Scolymus hispanicus* L.) which are collected and consumed by both people of Castelmezzano and Graecanic area but in Bologna. Conversely, species like *Sanguisorba minor* Scop., widely known as “*pampinela*”, and *Urtica* spp., are still very popular and used in Bologna’s territory, but were not present in the other two surveys [25, 26].

### Folk plant classification and folk names

Folk plant names were mentioned by the informants according to their own plant classification (*folk systematic*) in which the elementary unit is represented by a *folk generic*, also called *ethnospecies* (as defined by [29]), recognizable on the basis of differences in macro-morphology, habitat and use of the plant [30]. The present survey evidenced that, in several cases, more than one related plant species that cannot be distinguished by a non-expert were assimilated and identified as a single ethnospecies (*under differentiation*). It should be noted that wild food plants were usually collected at the rosette stage or as young shoots, when the plant lacks a flower, the most important botanical identification character. This is the case for folk plants commonly named “*frabbs”,* a term that equally refers to two species, *Sonchus asper* L. and *Sonchus arvensis* L., that are morphologically related but have a different leaf shape. As these two species have a similar taste, they were indiscriminately used and, therefore, share the same folk name. Analogously, the word “*radećć”* was equally assigned to *Cichorium intybus* L., *Sonchus* spp. L. and *Crepis* spp., a large group of plants of which the basal leaves were collected in the same period of the year (end of winter - beginning of spring, sometimes early autumn), eaten raw (tender leaves of young plants) or boiled (bigger older leaves collected in the late vegetative stage), and cooked in a similar way. In Dalmatia (southern Croatia), an analogous group of species (mainly *C. intybus* L. and *Crepis* spp.), belonging to Cichorioideae (Asteraceae family), are similarly called *“radić”* and collected as rosette basal leaves [31]. In general, plant names of *folk systematic* are not useful for botanical identification but rather associated to practical purposes and final use. In addition, plant folk names may be related to botanical characteristics, habitat, taste or the relationship between man and those plants. Our study pointed out several folk names which relate to a botanical character, such as “*strecapugno”*, which means “clenched in a fist”, referring to *C. vesicaria* subsp. *taraxacifolia* (Thuill) Thell (Table 1). In fact, local people know that this plant, after being cut from the ground, is going to rapidly close on itself and so they must clean it immediately after collection. *Piscialét* refers to the diuretic property of *T. officinale* Weber. As earlier mentioned, *C. intybus* L. was commonly indicated as “*radećć”* but also more specifically as “*radećć cavdagn”,* suggesting that this species belonging to the *radećć* category was mostly found along small country roads (*cavdagn*). Other examples of meaningful folk names are the above-mentioned *“erba brossca”*, which refers to the acid taste of *Rumex acetosa* L. leaves, and *“ciucchett”*, a onomatopoeic word that matches the popping sound produced when *Silene vulgaris* (Moench) Garcke flowers, which have a balloon-like capsule, are squeezed.

### Medicinal use of wild food plants

Eleven plant species were also mentioned as having therapeutic effects (Table 1). A few of these, after specific therapeutic preparation, were used as medicine without any direct relation to their alimentary use. *Lippia citriodora* Kuntze*, Salvia pratensis* L.*, Mentha* spp.*, Rosmarinus officinalis* L., all belonging to the Lamiaceae family, were used to make a decoction for digestive purposes. In addition, *L. citriodora* Kuntze leaf decoction was applied to treat muscular and articular pains after a trauma, *S. pratensis* L. was used for female genital problems (infusion) and as toothpaste (fresh leaves), while *R. officinalis* L. was utilized as a decongestant (fumigations). The flower infusion of *Crataegus monogyna* Jacq. was reported to be relaxing, to facilitate sleep and to be useful for heart problems. Other folk plants were reported to have therapeutic effects when part of the everyday diet. The wild species with the highest number of cited medicinal uses was *Urtica* spp., which if consumed with the diet or as cooking water, was reported to be refreshing, mineralizing and active against kidney problems. Moreover, *Urtica* cooking water was often used as shampoo to improve hair strength and shine and to eliminate dandruff. Other applications were as a remedy for arthritis (by rubbing fresh leaves on the aching areas of the body) and as insecticide (using macerated leaves). Some plants, in particular *T. officinale* Weber, *C. vesicaria* subsp. *taraxacifolia* (Thuill) Thell and *Sonchus* spp. were defined as *functional foods* [32] having depurative, blood cleaning and refreshing effects. Besides, *T. officinale* Weber, *C. vesicaria* subsp. *taraxacifolia* (Thuill) Thell (of which informants reported to also drink the cooking water) and *Crepis sancta* (L.) Babc. were in general reported to have diuretic and laxative actions so that these plants may all be considered *medicinal foods* [32]. Finally, *Sambucus nigra* L. consumed as jam was mentioned to relieve bone problems like rheumatisms.

### Perception and health impact of wild food plants

During the interviews, the informant’s perceptions regarding the impact of wild food plants on human nutrition and health, as well as the differences between wild, self-cultivated and large-scale cultivated edible plants were also investigated. All people interviewed perceived wild plants as being the healthiest for humans because they grow naturally without man’s intervention and, consequently, they are likely to contain the highest amounts of nutrients and beneficial substances. The respondents also perceived self-cultivated plants as better than those produced on a large-scale and purchased in stores because the exact process in this case was unknown.

### Taste of the collected wild food plants

Among the secondary metabolites produced by plants, phenolics, terpenes and alkaloids [33] are those that mainly contribute to the bitter, sour or astringent tastes [34–36]. These substances mostly accumulate in leaves and shoots, but also in flowers and roots and, among other effects, provide a defence against herbivorous predators by making the plant unpalatable [34]. Although potentially beneficial to human health in small doses, many of such compounds are, in fact, toxic [37]. Among the previously listed wild plants (Table 1), those reported to be the most bitter were *Clematis vitalba* L., *T. officinale* Weber and *C. vesicaria* subsp*. taraxacifolia* (Thuill) Thell, with the different degree of bitterness depending on individual perception. A wild edible plant with a particular strong flavour similar to arugula is *Diplotaxis tenuifolia* L. (DC) that, for this reason, was always used in combination with other vegetables. Instead, a delicate sweet flavour, also appreciated by children (often reluctant to eat wild greens because of their bitterness), was reported for *Silene vulgaris* (Moench) Garcke and for *Valerianella locusta* L. (Laterrade) which, therefore, were also eaten raw in salads (Table 1, Figure 3).

## Conclusions

The present study was aimed at documenting the traditional knowledge on wild food plants in a study area belonging to the province of Bologna (Emilia-Romagna region, Italy) (Figure 1) that had not been previously investigated by any other ethnobotanical research performed in Italy. The obtained results may allow to preserve part of the local cultural heritage that seems to be quickly disappearing along with the people, some of them very elderly, who still retain this type of knowledge. The popular traditions regarding wild food plants of the territory of Bologna are, in fact, being progressively lost because they are not handed down to new generations anymore, so that today young people do not acquire any information regarding wild edibles that characterized the diet of their forebears. Presumably, the high rate of immigration and the high level of urbanization also greatly contributed to the erosion of TLK. In particular the immigration phenomenon (from other areas of Italy and from abroad) has led to a mixture of traditions related to the use of wild food plants that partially overlap and influence each other. The present survey revealed that, in spite of the loss of TLK, the use of wild food plants in the area of Bologna is being revaluated today because these plants are perceived as healthy and represent the preservation of biodiversity as well as a way of getting back to nature.

In the era of large-scale distribution, which has generally led to a decrease in food quality, the interest in wild edibles is increasingly gaining media attention. In Italy and in many other European countries, it is possible to find guide books, workshops, and new culinary vogues associated with wild edible plants. A great impulse to this increased interest has also been given by the gastronomy elite, always in search for new stimuli, culinary experiences and healthy food, but also by agritourism farms and local rural restaurants desirous to put dishes of the traditional culinary heritage on their menus. Our contribution in preserving local knowledge and traditions will hopefully reinforce this new growing trend to become a habit, so as to enrich the local diet with new “old traditional” foods beneficial for human health.

## Electronic supplementary material

Additional file 1: **Questionnaire form Guidelines followed during the semi-structured interviews of the ethnobotanical survey.** (PDF 79 KB)
