# Supplementary material for: Wild food plants traditionally consumed in the area of Bologna (Emilia Romagna region, Italy)
Source: J Ethnobiol Ethnomed. 2014 Sep 25;10:69. doi: 10.1186/1746-4269-10-69 (PMC4189172; doi:10.1186/1746-4269-10-69)
Supplement: Supplementary file 1 — Additional file 1: Questionnaire form Guidelines followed during the semi-structured interviews of the ethnobotanical survey. (PDF 79 KB) [file 13002_2014_434_MOESM1_ESM.pdf]

Questionnaire form for ethnobotanical interviews

University of Bologna
Department of Biological, Geological and Environmental Sciences, University of Bologna,
Via Irnerio 42, 40126 Bologna, Italy

Identity of the interviewed person

Name:
Surname:
Residence:
Gender: female ☐ male ☐
Age:

Place of formation of the traditional knowledge

Place of birth:

Childhood place:
Teens age place:
Adult age place:

Wild food plants

Do you use wild plants as food? Yes ♩ No ☐
In the past ☐Nowadays ☐

Which ones? ➔ *freely recall all the used wild food plants*

Wild food plant mentioned:
Folk name

In which period of the year do you collect this plant?

Which parts of plant do you use?

How do you cook it?

During the collection period, how often do you eat it?
Once a day ☐
Once a week ☐
Once a month ☐
Once a year ☐
Never at present time, only in the past ☐
I've never eaten it, I have only heard about it ☐

Taste

What does it taste like?
Sweet ☐ Bitter ☐Acid ☐Salty ☐Astringent ☐
How does it looks like ________________

Do your sons like it? Yes ☐ No ☐ Do your grandchildren like it? Yes ☐No ☐

Functional foods/ Medical foods

Do you use any mentioned wild food plant as medicine?

Do the used wild food plants have any beneficial effects on your health?
If yes, describe these effects in detail.

Do the used wild food plants have adverse effects?
If yes, which ones?

Do you think they can have any impact on your life and your health?

Do these plants relieve the symptoms of any disease?
If yes, which ones?
